# Risk factors for the progression of finger interphalangeal joint osteoarthritis: a systematic review

**DOI:** 10.1007/s00296-020-04687-1

**Published:** 2020-08-24

**Authors:** Karishma Shah, Xiaotian Yang, Jennifer C. E. Lane, Gary S. Collins, Nigel K. Arden, Dominic Furniss, Stephanie R. Filbay

**Affiliations:** 1 Nuffield Department of Orthopaedics, Rheumatology and Musculoskeletal Sciences, Botnar Research Centre, University of Oxford, Oxford, UK; 2 Department of Rehabilitation Medicine, West China Hospital, Sichuan University, Chengdu, China; 3 Nuffield Department of Orthopaedics, Rheumatology and Musculoskeletal Sciences, Centre for Statistics in Medicine, University of Oxford, Oxford, UK; 4 National Institute for Health Research Oxford Biomedical Research Centre, John Radcliffe Hospital, Oxford, UK; 5 Nuffield Department of Orthopaedics, Rheumatology and Musculoskeletal Sciences, Centre for Sport, Exercise and Osteoarthritis Research Versus Arthritis, University of Oxford, Oxford, UK; 6 Department of Physiotherapy, Centre for Health Exercise and Sports Medicine, University of Melbourne, Melbourne, Australia

**Keywords:** Hand interphalangeal joint, Osteoarthritis, Risk factors, Disease progression

## Abstract

Progressive hand interphalangeal joint (IPJ) osteoarthritis is associated with pain, reduced function and impaired quality of life. However, the evidence surrounding risk factors for IPJ osteoarthritis progression is unclear. Identifying risk factors for IPJ osteoarthritis progression may inform preventative strategies and early interventions to improve long-term outcomes for individuals at risk of IPJ osteoarthritis progression. The objectives of the study were to describe methods used to measure the progression of IPJ osteoarthritis and identify risk factors for IPJ osteoarthritis progression. MEDLINE, EMBASE, Scopus, and The Cochrane Library were searched from inception to 19th February 2020 (PROSPERO CRD42019121034). Eligible studies assessed potential risk factor/s associated with IPJ osteoarthritis progression. Risk of bias was assessed using a modified QUIPS Tool, and a best evidence synthesis was performed. Of eight eligible studies, all measured osteoarthritis progression radiographically, and none considered symptoms. Eighteen potential risk factors were assessed. Diabetes (adjusted mean difference between 2.06 and 7.78), and larger finger epiphyseal index in males (regression coefficient *β* = 0.202) and females (*β* = 0.325) were identified as risk factors (limited evidence). Older age in men and women showed mixed results; 13 variables were not risk factors (all limited evidence). Patients with diabetes and larger finger epiphyseal index might be at higher risk of radiographic IPJ osteoarthritis progression, though evidence is limited and studies are biased. Studies assessing symptomatic IPJ osteoarthritis progression are lacking.

**Electronic supplementary material:**

The online version of this article (10.1007/s00296-020-04687-1) contains supplementary material, which is available to authorized users.

## Introduction

Osteoarthritis is one of the leading causes of worldwide disability [1], and, in the USA alone, carries a cost of $10 billion just from economic loss [2]. Hand osteoarthritis is one of the most common types of radiographic osteoarthritis [1]. Hand osteoarthritis also presents in a younger population than osteoarthritis at other joints, with a prevalence of 3% in men and 8% in women aged 45–64 years [3, 4]. It is considered a chronic disease, with some cases progressing and the prevalence increasing to 5% in men and 9% in women aged 65–74 years [5]. Symptomatic treatment for progressive hand osteoarthritis is limited, with patients often requiring surgical management, such as arthrodesis or arthroplasty [6].

Measuring progressive hand osteoarthritis is difficult, with no consensus for defining or quantifying worsening of disease [7]. The Osteoarthritis Research Society International (OARSI) 2006 Task Force described hand osteoarthritis progression as being joint specific, whereby osteoarthritis in one hand joint evolves independently from other hand joints [8]. However, analysis from a large cohort study suggests there are patterns of symmetry, osteoarthritis clustering by row (across distal interphalangeal joints (DIPJs) or across proximal interphalangeal joints (PIPJs)), and clustering by ray (within a finger) also exist [9]. There is also poor correlation between radiographic and symptomatic disease [10, 11].

The aetiology for the progression of hand osteoarthritis is also poorly understood, and therefore identifying patients at highest risk for needing surgical management is limited. When managing hip and knee osteoarthritis surgically, shared decision making between clinicians and patients has been shown to be beneficial [12]. In the hand, a better understanding of whether a patient is at increased risk of progressive disease would help to inform shared decision making. In particular, it would enable earlier investigations, more personalised treatment pathways, and targeted interventions for prevention and treatment. These priorities have been highlighted by the recent Commission on the Future of Surgery [13]. Similarly, being able to identify patients with osteoarthritis who will not progress will prevent the over-investigation and excessive medical treatment of these patients. A review has found that abnormal scintigraphy scans, higher Australian/Canadian Hand Osteoarthritis Index (AUSCAN) scores, number of osteoarthritis joints at baseline, more pain, and nodal osteoarthritis were risk factors for the progression of radiographic or clinical hand osteoarthritis [14]. However, this review combined interphalangeal joint (IPJ) and base of thumb [first carpometacarpal joint (CMCJ)] osteoarthritis under the umbrella of ‘hand osteoarthritis.’ Finger IPJ and first CMCJ osteoarthritis are now thought to be different subsets of the disease, with different risk factors, pathophysiology and patterns of progression [15].

Therefore, the primary aim of this systematic review is to identify risk factors for the progression of finger IPJ osteoarthritis. The secondary aim is to describe the measurements used to define the progression of IPJ osteoarthritis.

## Methods

The reporting of this systematic review followed the Preferred Reporting Items for Systematic reviews and Meta-Analysis (PRISMA) Statement [16]. The protocol was prospectively registered on PROSPERO [17] (CRD42019121034).

### Search strategy

The search strategy was constructed with the assistance of a specialist health-care librarian. The search was conducted in four electronic databases: (1) Medline by Ovid, (2) Embase by Ovid, (3) Scopus, (4) the Cochrane library. The search string included a range of search terms for (1) hands and fingers, (2) osteoarthritis, and (3) progression, and was amended for each database (Electronic Supplementary Material 1). The PICOS tool [18] was used to frame the search strategy as follows: population: adults with IPJ osteoarthritis, intervention/prognostic factor: potential risk factor(s) for IPJ osteoarthritis progression, comparison: no exposure to the risk factor(s), outcome: progression of IPJ osteoarthritis, study type: quantitative methodology. The search was conducted on 17th October 2018 and duplicates were removed. The search was updated on 19th February 2020. The reference lists of all eligible articles were manually assessed for additional studies. Rayyan QCRI Tool was used to import all papers [19].

Two groups of reviewers (Group 1: KS, Group 2: XY and JCEL) independently screened titles and abstracts for eligibility. Any articles with insufficient title or abstract information were referred for full text review. Articles for which the full text was not available were requested directly from the authors. Any disagreements in eligibility assessment was resolved at a consensus meeting by a third reviewer (SRF).

### Study eligibility criteria

Studies were considered eligible if they (a) included participants with evidence of radiographic or clinical IPJ osteoarthritis at baseline; (b) the participants were followed up for at least 1 year (as it has been shown that progression of radiographic hand osteoarthritis can be detected over a 1 year time frame [20]); (c) IPJ osteoarthritis (separate from first CMCJ osteoarthritis) progression was measured at follow-up, using radiographic and/or symptomatic criteria (IPJ osteoarthritis progression was defined as an increase in radiographic or symptomatic criteria/score at follow-up compared to baseline); (d) the association between a potential risk factor and the progression of IPJ osteoarthritis was investigated at follow-up.

Case reports were excluded. Letters to editors might contain important information about studies, such as new information or discussions of further weaknesses of original studies [21]. Therefore, letters to editors which exist in the context of original studies, included in our review, were examined to inform the risk of bias assessment and as additional sources of information [22]. Conference abstracts are considered to have high variability in terms of data reliability, accuracy and detail, and therefore these were excluded [21, 23].

Studies of inflammatory arthritis, erosive arthritis, with participants under the age of 18 years (to avoid confounding by juvenile arthritis), and studies where IPJ osteoarthritis results could not be separated from other joints including the first CMCJ, and were not provided on request of the corresponding author within 2 months were excluded. Animal, cadaver, and cell studies were excluded. Articles not in English, and articles which lacked accessible full texts (online or in paper copy throughout the UK, or after requesting them from the corresponding author with no reply within 2 months) were excluded.

### Data extraction

One reviewer (KS) independently extracted participant demographics (e.g. age and sex), study characteristics (e.g. study design), the potential risk factor/s assessed, effect measure and size/s and the definition/s used to measure osteoarthritis progression. A potential risk factor was defined as any factor investigated for an association with IPJ osteoarthritis progression.

If data was reported at multiple time points, results from all time points were extracted. If articles or supplementary material did not contain sufficient data, the corresponding author was contacted to request additional data, with a 2 month turnaround policy. For any articles which reported data from a study described in detail elsewhere, the source of the data was retrieved and data extracted as appropriate. Data extraction was input into a Microsoft Excel file and cross-checked by a second independent reviewer (XY).

### Risk of bias assessment

Two independent reviewers (KS and XY) rated the risk of bias of included studies using a modified version of the Quality in Prognosis Studies (QUIPS) risk of bias tool [24] (Electronic Supplementary Material 2). The following five domains were assessed: (1) study participation, (2) study attrition, (3) prognostic factor measurement, (4) outcome measurement, (5) statistical analysis and reporting [24]. We excluded the domain assessing ‘Confounding factors’, as confounders can themselves be considered to be prognostic factors, and thus the term ‘confounders’ is a misnomer in prognostic factor studies [25]. As there is currently limited established literature in the field of IPJ osteoarthritis progression, any ‘confounder’ identified in the literature was treated as a potential risk factor for this review. Each domain was given an overall score of ‘low’, ‘moderate’ or ‘high’ risk of bias (Electronic Supplementary Material 2). The overall risk of bias of a study was classified by examining the risk of bias in each of the five domains. If one or more domains were classified as having high risk of bias, then this study was classified as having an overall high risk of bias [24, 26–28]. If three or more domains were classified as having a moderate risk of bias, then this study was classified as having an overall moderate risk of bias [24, 26, 27, 29]. If all domains were classified as having a low risk of bias, or less than three domains had a moderate risk of bias, then this study was classified as having an overall low risk of bias [28, 30]. Any disagreement between reviewers was discussed at a consensus meeting with a third reviewer (SRF).

### Analysis and best evidence synthesis

Risk factors for all definitions of IPJ osteoarthritis progression were identified, followed by a subgroup analysis for DIPJ and PIPJ separately. If studies were homogenous with regard to study populations, potential risk factors assessed, effect measures used, and measurements of IPJ osteoarthritis progression, a pooled meta-analysis was considered using Review Manager software [31], and the Grading of Recommendations, Assessment, Development and Evaluation (GRADE) approach was used to assess the quality of evidence [32].

If studies were heterogeneous, we chose not to report effect measures of different types and instead used a qualitative narrative summary. The association between a potential risk factor and IPJ osteoarthritis progression was categorised as:

- A risk factor: positive effect measure.
- Not a risk factor: negative effect measure; or, no statistical association.
- Conflicting evidence: effect measures not in the same direction.

A best evidence synthesis was used to summarise the data for each potential risk factor assessed [33–36]. The criteria were applied sequentially. If multiple analyses were performed within one study, the consistent findings approach described below was applied to the study to decide whether it showed consistent or mixed evidence. This was then used to calculate the overall best evidence synthesis across studies.

- Consistent evidence: ≥ 75% of studies reported the same direction of effect (either positive or negative/no association).
- Mixed evidence: < 75% of analyses reported the same direction of effect.

If consistent evidence was found, the strength of evidence was assessed:

(i) Strong evidence: > 2 studies with low risk of bias.
(ii) Moderate evidence: 1 study with low risk of bias. and 1 other study; or: > 2 studies with moderate or high risk of bias.
(iii) Limited evidence with low risk of bias: 1 study with low risk of bias.
(iv) Limited evidence: ≤ 2 studies with moderate or high risk of bias.

## Results

### Studies included

**Fig. 1:**
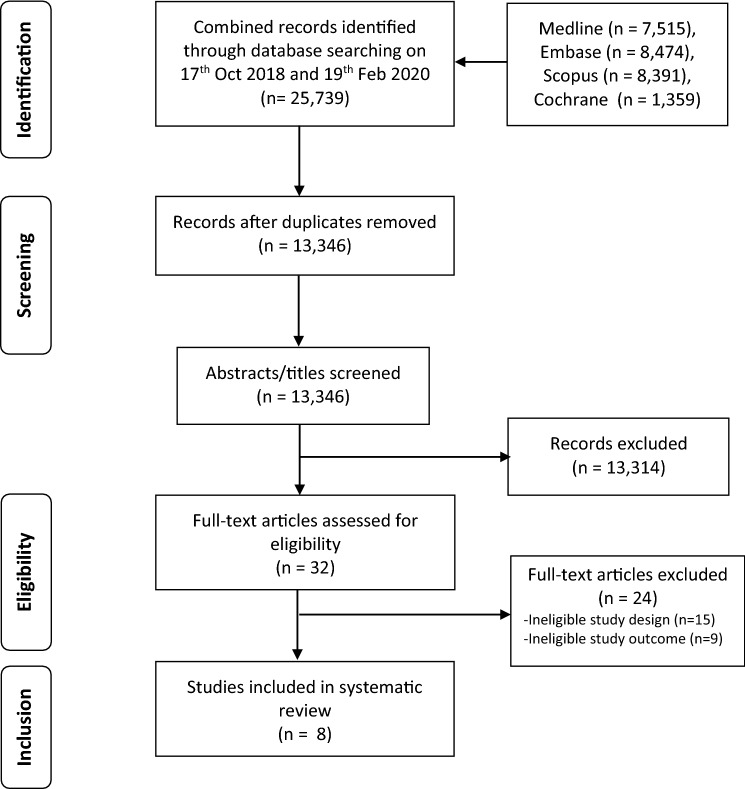
Preferred Reporting Items for Systematic Reviews and Meta-analysis (PRISMA) flowchart of study selection

Combining results from the search in October 2018 and the updated search in February 2020, 25, 739 titles were identified through the search strategy, with 13,346 remaining after removal of duplicates. After screening titles and abstracts, the full text of 32 articles was evaluated, and eight articles met the inclusion criteria (Fig. 1). No additional articles were found by reviewing the reference lists of eligible studies.

### Study characteristics

Eight prospective cohort studies were included [37–44] (Table 1). Five studies included men and women [40–44], whilst three studies included only men [37–39]. The smallest study included 177 participants [37], whilst the largest study included 5560 participants [40]. The shortest follow-up period was a mean of 2.28 years [39] and the longest follow-up was reported as a mean (standard deviation) of 23.5 (3.3) years [37].

**Table 1:** Characteristics of studies investigating risk factors for the progression of finger interphalangeal joint osteoarthritis

Authors	Population	Length of follow-up (years)	Age (years) (mean)	Female (%)	Inclusion criteria	Exclusion criteria	*N* (*n*)	Criteria for IPJ OA progression	Risk factor assessed
Plato et al. [35]	White middle class volunteers participated in the BLSA in the USA	Group 1: 0–3, group 2: 4–7, group 3: 8–11, group 4: 12–16	NS	0	NS	NS	478 (NS)	Increase by ≥ 1 grade from the highest KL [41] grade at baseline in any DIPJ	Older age in men
Kallman et al. [33]	White middle class volunteers who participated in the BLSA in the USA	≥ 20 (age < 60 years); ≥ 14 (age ≥ 60 years)	NS	0	NS	Maximum KL score (4) at baseline (per patient); Not specified	177 (177)	Increase by ≥ 1 grade from the highest KL [41, 42] grade at baseline in any PIPJ	Older age in men
Busby et al. [34]	White middle class volunteers who participated in the BLSA in the USA	5–16.3	NS	0	NS	Joints with KL score of 4 at baseline	386 (NS)	Outcome 1: increase by ≥ 1 grade from the highest KL [42] grade at baseline in any IPJ (DIPJ and PIPJ assessed separately) Outcome 2: increase in number of IPJs with KL [42] grade ≥ 2 (DIPJ and PIPJ assessed separately)	Older age in men
Kalichman et al. [39]	Chuvashians; Village; Randomly recruited	8	Men: 45.3, Women: 49.7	52	NS	NS	263 (263)	Increase in number of IPJs with KL [42] grade ≥ 2 (DIPJ and PIPJ assessed separately)	Alcohol, anthropometric features, familial relationship, gender (female), older age in men, older age in women, smoking
Kalichman et al. [40]	Chuvashians; Village; Randomly recruited	8	Men: 47.4, women: 50.9	46	NS	Bone disease, amenorrhoea, hormone replacement therapy, steroids	557 (513)	Increase by ≥ 1 grade in a cumulative KL [38] sum score (2nd, 3rd and 4th, PIPJs)	Epiphyseal index (larger)
Hoeven et al. [36]	Rotterdam	10	Men: 67.5, women: 68.6	58	≥ 55 years, living for ≥ 1 year in Ommoord, knee, hip, hand X-rays	No X-rays, rheumatoid, fractures	5650 (2442)	Increase by ≥ 1 KL [41] grade in ≥ 1 IPJ, if ≥ 1 IPJ had KL [41] grade ≥ 2 at baseline (DIPJ and PIPJ assessed separately)	Atherosclerosis
Haugen et al. [37]	USA; Hospital study sites	4	58.4	58	NS	Systemic inflammatory arthritis, bilateral end stage knee OA, inability to walk without aids, contraindication to MRI	994 (994)	Increase by ≥ 1 grade in a cumulative modified KL [42, 53] sum score (DIPJ and PIPJ assessed together)	Alcohol (higher intake), BMI (higher)—at age 25, BMI (higher)—current, smoking, waist circumference (higher)
Marshall et al. [38]	From CASHA and CASK cohorts; GP community	7	60.5	60	Age 50–69 years at baseline, reported hand pain in last month	Inflammatory arthritis, all hand joints affected with KL ≥ 2 at baseline, deaths/untraceable/address unknown, severe/terminal illness	706 (388)	Outcome 1: Increase by ≥ 1 grade in a cumulative KL [43] sum score (DIPJ and PIPJ assessed together) Outcome 2: increase in number of IPJs with KL [43] grade ≥ 2 (DIPJ and PIPJ assessed together)	BMI (higher)—current, diabetes type 2/impaired fasting glucose, dyslipidaemia, hypertension, number of metabolic factors (higher)

*BLSA* Baltimore Longitudinal Study of Aging, *BMI* body mass index, *CASHA* Clinical Assessment Studies of the Hand, *CASK* Clinical Assessment Studies of the Knee, *DIPJ* distal interphalangeal joint, *GP* general practice, *IPJ* interphalangeal joint, *KL* Kellgren–Lawrence atlas, *PIPJ* proximal interphalangeal joint, *N* number at baseline, *n* number at follow-up, *NS* not specified, *OA* osteoarthritis, *USA* United States of America, *X-rays* plain film radiographs

### Risk of bias

Seven studies were rated as having overall high risk of bias [37–39, 41–44], and one study was of moderate risk of bias [40] (Table 2). ‘Study participation’ was of high risk of bias in four studies due to studies not adequately reporting recruitment periods and places of recruitment [37, 39, 41, 44]. In the ‘Study attrition’ domain, Plato et al. and Kallman et al. did not clearly report response rates and reasons for participants with loss to follow-up [37, 39], whilst Haugen et al. and Marshall et al. had less than 80% response rates and also did not report reasons for loss to follow-up [41, 42]. When assessing the ‘Statistical analysis and reporting’ domain, it was found that Plato et al., Busby et al., and Kalichman et al. did not provide effect measures, but only reported *p* values or stated whether results were ‘significant or not significant’ [38, 39, 43].

**Table 2:** Risk of bias for studies assessing potential risk factors for the progression of finger interphalangeal joint osteoarthritis, assessed using a modified Quality in Prognosis Studies (QUIPS) tool

Authors	Biases^a^					Overall risk of bias
	1	2	3	4	5	
Plato et al. [35]	High	High	Moderate	Moderate	High	High
Kallman et al. [33]	High	High	Low	Low	Moderate	High
Busby et al. [34]	Moderate	Moderate	Low	Moderate	High	High
Kalichman et al. [39]	Moderate	Low	Moderate	Moderate	High	High
Kalichman et al. [40]	High	High	Moderate	Low	Moderate	High
Hoeven et al. [36]	Moderate	Low	Moderate	Low	Moderate	Moderate
Haugen et al. [37]	High	High	Moderate	Moderate	Moderate	High
Marshall et al. [38]	Moderate	High	Low	Moderate	Low	High

^a^Biases from modified Quality in Prognosis Studies (QUIPS) tool: (1) study participation; (2) study attrition; (3) prognostic factor measurement; (4) outcome measure; (5) statistical analysis and reporting

### Measurements for the progression of finger interphalangeal joint osteoarthritis

All studies assessed osteoarthritis radiographically, using a version of the Kellgren and Lawrence (KL) classification [45, 46] (Table 1). Three studies measured IPJ osteoarthritis progression as a ≥ 1 grade increase from the highest KL grade at baseline [37–39]; three studies measured it as an increase in the total number of IPJs with KL grade ≥ 2 [38, 42, 43]; one study measured progression as a ≥ 1 grade KL increase in a ≥ 1 IPJ [40]; and three studies measured it as ≥ 1 grade increase in a cumulative KL sum score [41, 42, 44]. No studies measured osteoarthritis progression through a deterioration in symptomatic scoring.

### Risk factors for the progression of finger interphalangeal joint osteoarthritis

Eighteen potential risk factors were assessed, most commonly in one study only (effect measures shown in Electronic Supplementary Material 3). For potential risk factors assessed by more than one study, due to heterogeneity in the definitions of the risk factor/s, statistical tests, and osteoarthritis definitions, a best evidence synthesis was performed. Three risk factors were identified: diabetes type 2/impaired fasting glucose (IFG) [42]; and larger epiphyseal index (EI) in males [44], and in females [44] (all with limited evidence) (Table 3). Older age in men [37–39, 43] and in women [43] showed mixed results (Table 3).

**Table 3:** Potential risk factors for the progression of finger interphalangeal joint osteoarthritis, assessed using a best evidence synthesis

Consistent evidence for a risk factor				Consistent evidence for not being a risk factor				Mixed evidence
Strong evidence	Moderate evidence	Limited evidence with low risk of bias	Limited evidence	Strong evidence	Moderate evidence	Limited evidence with low risk of bias	Limited evidence	
Using all definitions of IPJ osteoarthritis progression								
			Diabetes/impaired fasting glucose [38]				Higher alcohol intake [37, 39]	Older age in men [33–35, 39]^a^
			Larger epiphyseal index in females [40]				Anthropometric features [39]	Older age in women [39]^a^
			Larger epiphyseal index in males [40]				Atherosclerosis [36]	
							Larger BMI—at age 25 years [37]	
							Larger BMI—current [37, 38]	
							Dyslipidaemia [38]	
							Familial relationship [39]	
							Gender (female) [39]	
							Gender (male) [39]	
							Hypertension [38]	
							Higher number of metabolic factors [38]	
							Smoking [37, 39]	
							Larger waist circumference [37]	
In DIPJs only								
			Older age in women [39]				Higher alcohol intake [39]	Gender (female) [39]
							Anthropometric features [39]	Older age in men [34, 39]
							Atherosclerosis [36]	
							Familial relationship [39]	
							Gender (male) [39]	
In PIPJs only								
			Larger epiphyseal index in females [40]				Higher alcohol intake [39]	Older age in men [33–35, 39]^a^
			Larger epiphyseal index in males [40]				Anthropometric features [39]	Older age in women [39]
							Atherosclerosis [36]	
							Familial relationship [39]	
							Gender (female) [39]	
							Gender (male) [39]	
							Smoking [39]	

*BMI* body mass index, *DIPJ* distal interphalangeal joints, *IPJ* interphalangeal joint, *PIPJ* proximal interphalangeal joints

^a^Conflicting results within one study

## Diabetes type 2/impaired fasting glucose (IFG)

Marshall et al. assessed diabetes type 2/IFG compared to not having these conditions in a total of 474 participants [42] (effect measures shown in Electronic Supplementary Material 3). In a complete case analysis, these conditions were associated with an increase by ≥ 1 grade in a cumulative KL [47] sum score for all IPJs [adjusted mean difference (95% confidence interval) 7.78 (1.13–14.43)] [42]. However, there was no association following multiple imputation [4.50 (− 0.26 to 9.25)] [42]. Diabetes type 2/IFG was associated with an increase in the number of IPJs with KL [47] grade ≥ 2 following multiple imputation and complete case analysis [2.06 (0.25–3.87) and 3.35 (1.08–5.62), respectively] [42].

## Large finger epiphyseal index (EI)

Kalichman et al. investigated larger EI in 177 participants [44] (Electronic Supplementary Material 3). A positive association was found in both males (multiple regression coefficient, *β* = 0.202; 95% CI not reported) and females (*β* = 0.325; 95% CI not reported), between larger EI and IPJ osteoarthritis progression (measured as an increase by ≥ 1 grade in a cumulative KL [38] sum score for PIPJs in the assessed digits).

### DIPJ and PIPJ subgroup analysis

In the DIPJ subgroup analysis, eight potential risk factors were assessed, and only older age in women was found to be a risk factor (correlation coefficient 0.20) [43] (limited evidence) (Table 3). In the PIPJ subgroup analysis, 11 potential risk factors were assessed, and larger EI in males (*β* = 0.202; 95% CI not reported) and females (*β* = 0.325; 95% CI not reported) were identified as risk factors [44] (limited evidence for both) (Table 3).

## Discussion

Osteoarthritis is one of the largest health-care burdens, and radiographic hand osteoarthritis is highly prevalent, affecting more than one out of five adult Americans [48]. Osteoarthritis is considered to be progressive in some cases. However, there is no unified method to measure the progression of hand osteoarthritis, and IPJ osteoarthritis is now considered to be a different disease subset from first CMCJ osteoarthritis. As IPJ osteoarthritis progresses, it can be treated surgically, and there are currently no disease-modifying drugs. Risk factors which increase the chance of IPJ osteoarthritis progression in patients have been studied in the literature. We identified eight studies (seven high risk of bias) investigating potential risk factors for the progression of finger IPJ osteoarthritis [37–44]. All studies measured osteoarthritis progression radiographically, using a version of the KL classification system [45, 46]. Our review found that patients with diabetes/IFG [42], and both male and females with a larger finger EI [44], are at increased risk of IPJ osteoarthritis progression (limited evidence), whilst older age in men [37–39, 43] and in women [43] showed mixed evidence. Results were largely similar when DIPJ and PIPJ osteoarthritis when assessed separately.

The KL classification system [45, 46] was used to measure osteoarthritis progression by all studies [37–44, 49]. The KL classification system [45, 46] is a sensitive method for measuring the progression of radiographic hand osteoarthritis over a 1-year time frame [20]. All of the studies included in this review were longitudinal studies, and the shortest follow-up period had a mean of 2.28 years [39]. Therefore, all studies would have adequately detected any radiographic IPJ osteoarthritis progression. However, the definitions of each measure of progression varied across studies. Some studies measured an increase in KL grade [37–40], whilst others measured it as an increase in the number of joints with a particular KL grade [38, 40, 42, 43] and still other studies measured it as an increase in a cumulative KL sum score [41, 42, 44] (which is dependent on either an increase in KL grade of already affected joints, or an increase in the number of joints with a particular KL grade). The sensitivity of the KL classification system [45, 46] in detecting IPJ osteoarthritis progression measured in these different ways has not yet been investigated. Additionally, potential risk factors that occur at a localised joint level (such as joint trauma) could also be risk factors for isolated IPJ osteoarthritis progression. However, localised risk factors were not assessed by studies in this review. Further research is required to understand whether there are any joint-specific risk factors for IPJ osteoarthritis progression, and whether these might cause osteoarthritis to progress at one joint independently of other IPJs.

Diabetes/IFG was found to be a risk factor for IPJ osteoarthritis progression [42]. Marshall et al. suggest diabetes/IFG might be a risk factor for the progression of osteoarthritis due to hyperglycaemia [42]. Hyperglycaemia has been shown to induce reactive oxygen species and the production of cytokines, which result in joint inflammation and in the production of proteolytic enzymes that degrade cartilage [50]. However, in a Delphi study consisting of a panel of hand surgeons, the use of diabetic medication and abnormal fasting glucose were not identified as risk factors for finger IPJ osteoarthritis progression [51]. This suggests that though diabetes/IFG might have a relationship with IPJ osteoarthritis on a molecular level, in a clinical context the effect is not yet well recognised. Our results also found that larger finger EI is a risk factor for IPJ osteoarthritis progression [44]. In hip and knee osteoarthritis, larger cross-sectional areas in the femoral neck and proximal femoral shaft and in the tibial plateau, respectively, have also been described [52, 53]. Additionally, in knee osteoarthritis, a loss of articular cartilage coupled with larger bone epiphyseal area results in a change of loading and force across a joint, further contributing to the progression of osteoarthritis [54]. However, in the hands, and particularly the finger IPJs, the load across the joint is much lower, suggesting that there might be other mechanisms which contribute to the relationship between EI and IPJ osteoarthritis progression. Given the limited evidence reported in our systematic review, further high-quality studies are needed to assess this relationship.

The studies included in this systematic review are limited by their moderate/high risk of bias [37–39, 41–44]. This resulted in lower levels of evidence for each potential risk factor assessed. All studies included in this review used radiographic methods to measure osteoarthritis progression. However, there is poor correlation between radiographic and symptomatic osteoarthritis [10, 11], and studies assessing risk factors for the progression of symptomatic IPJ osteoarthritis are required. The time interval between baseline and follow-up measurements of osteoarthritis varied between studies, making it difficult to quantify the exact impact of a potential risk factor on osteoarthritis progression. Similarly, study populations varied with regard to age of participants, which could moderate the effect of other potential risk factors. All studies in this review are Phase 1 or 2 prognostic factor studies. Phase 3 studies are required to more adequately understand the effect of multiple risk factors on the progression of IPJ osteoarthritis and assess prognostic pathways [55, 56]. During data extraction, it became clear that studies used a variety of definitions to measure the progression of IPJ osteoarthritis. Therefore, a secondary aim of this review was established, to describe the criteria used to define the progression of IPJ osteoarthritis. This resulted in deviation from the study protocol registered on PROSPERO [17]. However, performing this analysis showed that there is great heterogeneity in the way IPJ osteoarthritis progression is defined, and further consensus work is needed to establish common criteria for defining disease progression. This review is limited by a small number of studies assessing each potential risk factor and meta-analyses could not be performed. Future work should also focus on standardising definitions of IPJ osteoarthritis progression, enabling harmonisation of datasets and pooling of study data.

## Conclusion

Few studies which assess potential risk factors for hand IPJ osteoarthritis progression exist, and most are of high risk of bias. In the literature, the progression of IPJ osteoarthritis is measured radiographically using the KL classification system [45, 46], and no studies on symptomatic osteoarthritis progression were identified. Diabetes/IFG and larger finger EI are risk factors for disease progression, though evidence is limited. A better understanding of risk factors is needed to inform the identification and management of patients with a high risk of IPJ osteoarthritis progression.

## Electronic supplementary material

Below is the link to the electronic supplementary material.

Supplementary file1 (DOCX 16 kb)

Supplementary file2 (DOCX 17 kb)

Supplementary file3 (XLSX 31 kb)

## Data Availability

Provided in the Electronic Supplementary Material.
