# Correction to: Risk factors for the progression of finger interphalangeal joint osteoarthritis: a systematic review

**DOI:** 10.1007/s00296-021-04823-5

**Published:** 2021-04-22

**Authors:** Karishma Shah, Xiaotian Yang, Jennifer C. E. Lane, Gary S. Collins, Nigel K. Arden, Dominic Furniss, Stephanie R. Filbay

**Affiliations:** 1 Nuffield Department of Orthopaedics, Rheumatology and Musculoskeletal Sciences, Botnar Research Centre, University of Oxford, Oxford, UK; 2 Department of Rehabilitation Medicine, West China Hospital, Sichuan University, Chengdu, China; 3 Nuffield Department of Orthopaedics, Rheumatology and Musculoskeletal Sciences, Centre for Statistics in Medicine, University of Oxford, Oxford, UK; 4 National Institute for Health Research Oxford Biomedical Research Centre, John Radcliffe Hospital, Oxford, UK; 5 Nuffield Department of Orthopaedics, Rheumatology and Musculoskeletal Sciences, Centre for Sport, Exercise and Osteoarthritis Research Versus Arthritis, University of Oxford, Oxford, UK; 6 Department of Physiotherapy, Centre for Health Exercise and Sports Medicine, University of Melbourne, Melbourne, Australia

## Correction to: Rheumatology International (2020) 40:1781–1792 10.1007/s00296-020-04687-1

In the original article published, the references are cited incorrectly in Tables 1, 2 and 3. The correct information is given below:

The correct citation number for the study Plato et al. is reference number 39: Plato CC, Norris AH. Osteoarthritis of the hand: longitudinal studies. Am J Epidemiol. 1979;110(6):740–746.

The correct citation number for the study Kallman et al. is reference number 37: Kallman DA, et al. The longitudinal course of hand osteoarthritis in a male population. Arthritis Rheum. 1990;33(9):1323–1332.

The correct citation number for the study Busby et al. is reference number 38: Busby J, et al. A longitudinal study of osteoarthritis of the hand: the effect of age. Ann Hum Biol. 1991;18(5):417–424.

The correct citation number for the study Kalichman et al. is reference number 43: Kalichman L, et al. Repeated measurement study of hand osteoarthritis in an apparently healthy Caucasian population. Am J Hum Biol. 2005;17(5):611–621.

The correct citation number for the study Kalichman et al. is reference number 44: Kalichman L, et al. Epiphyseal expansion in hand bones: association with age, sex, and hand osteoarthritis. Osteoarthr Cartil. 2008;16(5):560–565.

The correct citation number for the study Hoeven et al. is reference number 40: Hoeven TA, et al. Association of atherosclerosis with presence and progression of osteoarthritis: the Rotterdam Study. Ann Rheum Dis. 2013;72(5):646–651.

The correct citation number for the study Haugen et al. is reference number 41: Haugen IK, et al. The prevalence, incidence, and progression of hand osteoarthritis in relation to body mass index, smoking, and alcohol consumption. J Rheumatol. 2017;44(9):1402–1409.

The correct citation number for the study Marshall et al. is reference number 42: Marshall M, et al. Metabolic risk factors and the incidence and progression of radiographic hand osteoarthritis: a population-based cohort study. Scand J Rheumatol. 2019;48(1):52–63.

