# Supplementary material for: Risk factors for the progression of finger interphalangeal joint osteoarthritis: a systematic review
Source: Rheumatol Int. 2020 Aug 24;40(11):1781–92. doi: 10.1007/s00296-020-04687-1 (PMC7519919; doi:10.1007/s00296-020-04687-1)
Supplement: Supplementary file 1 — Supplementary file1 (DOCX 16 kb) [file 296_2020_4687_MOESM1_ESM.docx]

**a)Search strategy for Medline by OVID**

| 1 hand/ or fingers/ or metacarpus/ or wrist/ (74741)  2 hand joints/ or finger joint/ or exp wrist joint/ (15060)  3 (finger* or hand or hands or interphalangeal*).ti,ab. (483537)  4 1 or 2 or 3 (520501)  5 OSTEOARTHRITIS/ (34319)  6 exp arthritis/ (241105)  7 (osteoarthrit* or osteoartrit* or osteo-arthrit* or osteo-artrit* or arthriti* or artriti* or arthros* or artros* or osteoarthros* or osteoartros* or OA).ti,ab. (250268)  8 5 or 6 or 7 (344300)  9 exp disease progression/ (158445)  10 exp prognosis/ (1455362)  11 INCIDENCE/ (234958)  12 exp risk/ (1088415)  13 (increas* or worse* or deteriorat* or caus* or predict* or prognos* or precipitat* or inciden* or frequen* or rate* or occurr*).ti,ab. (10587761)  14 9 or 10 or 11 or 12 or 13 (11477056)  15 4 and 8 and 14 (9866)  16 exp animals/ not humans.sh. (4505268)  17 15 not 16 (9495)  18 limit 17 to (case reports or comment or editorial or letter) (1094)  19 17 not 18 (8401)  20 exp Pediatrics/ (54039)  21 (Infan* or newborn* or new-born* or perinat* or neonat* or baby* or babies or toddler* or minors* or boy or boys or boyhood or girl* or kid or kids or child* or adolescen* or juvenil* or youth* or teen* or "under*age*" or pubescen* or pediatric* or paediatric* or peadiatric* or school* or prematur* or preterm*).ti,ab. (2442590)  22 20 or 21 (2451144)  23 19 not 22 (7956)  24 23 (7956)  25 limit 24 to english language (6885)  26 limit 25 to rd=19550101-20180711 (6558) |
| --- |

**b)Search strategy for Embase by OVID**

| 1 hand joint/ or finger joint/ (3173)  2 hand/ or hand muscle/ or hand palm/ or palmar fascia/ or thenar/ (32790)  3 finger/ or index finger/ or little finger/ or middle finger/ or ring finger/ (25869)  4 (finger* or hand or hands or interphalangeal*).ti,ab. (620609)  5 1 or 2 or 3 or 4 (635769)  6 exp arthritis/ (413385)  7 (osteoarthrit* or osteoartrit* or osteo-arthrit* or osteo-artrit* or arthriti* or artriti* or arthros* or artros* or osteoarthros* or osteoartros* or OA).ti,ab. (345212)  8 6 or 7 (498357)  9 exp disease course/ (2939814)  10 prognosis/ (528232)  11 incidence/ (325251)  12 exp risk/ (2195113)  13 (increas* or worse* or deteriorat* or caus* or predict* or prognos* or precipitat* or inciden* or frequen* or rate* or occurr*).ti,ab. (13309841)  14 9 or 10 or 11 or 12 or 13 (14973145)  15 5 and 8 and 14 (16374)  16 limit 15 to (conference abstract or conference paper or editorial or letter or reports) (6382)  17 limit 15 to conference abstracts (6097)  18 16 or 17 (6397)  19 15 not 18 (9977)  20 exp pediatrics/ (94751)  21 (Infan* or newborn* or new-born* or perinat* or neonat* or baby* or babies or toddler* or minors* or boy or boys or boyhood or girl* or kid or kids or child* or adolescen* or juvenil* or youth* or teen* or "under*age*" or pubescen* or pediatric* or paediatric* or peadiatric* or school* or prematur* or preterm*).ti,ab. (2922223)  22 20 or 21 (2936353)  23 19 not 22 (9333)  24 23 not ((exp animal/ or nonhuman/) not exp human/) (8905)  25 limit 24 to english language (7329) |
| --- |

**c) Search strategy for Scopus**

| 1. TITLE-ABS-KEY ( ( finger* OR hand OR hands OR interphalangeal*) )  2. TITLE-ABS-KEY ( osteoarthrit* OR osteoartrit* OR osteo-arthrit* OR osteo-artrit* OR arthriti* OR artriti* OR arthros* OR artros* OR osteoarthros* OR osteoartros* OR OA )  3. TITLE-ABS-KEY ( risk* OR increas* OR worse* OR deteriorat* OR caus* OR predict* OR prognos* OR precipitat* OR inciden* OR frequen* OR rate* OR occurr* )  4. #1 and #2 and #3  5. TITLE-ABS-KEY ( infan* OR newborn* OR new-born* OR perinat* OR neonat* OR baby* OR babies OR toddler* OR minors* OR boy OR boys OR boyhood OR girl* OR kid OR kids OR child* OR adolescen* OR juvenil* OR youth* OR teen* )  6. TITLE-ABS-KEY ("under*age*" OR pubescen* OR pediatric* OR paediatric* OR peadiatric* OR school* OR prematur* OR preterm* )  7. #5 OR #6  8. #4 AND NOT #7  9. 8 AND ( EXCLUDE ( DOCTYPE , "cp" ) OR EXCLUDE ( DOCTYPE , "le" ) OR EXCLUDE ( DOCTYPE , "ed" ) OR EXCLUDE ( DOCTYPE , "cr" ) ) AND ( LIMIT-TO ( EXACTKEYWORD , "Human" ) ) AND ( LIMIT-TO ( LANGUAGE , "English" ) ) |
| --- |

**d)Search strategy for The Cochrane Library**

| 1 MeSH descriptor: [Fingers] explode all trees 825  #2 MeSH descriptor: [Wrist] explode all trees 310  #3 MeSH descriptor: [Hand] this term only 1462  #4 MeSH descriptor: [Hand Joints] this term only 61  #5 MeSH descriptor: [Finger Joint] explode all trees 109  #6 MeSH descriptor: [Wrist Joint] explode all trees 234  #7 finger* or hand or hands or interphalangeal* 31333  #8 #1 or #2 or #3 or #4 or #5 or #6 or #7 31640  #9 MeSH descriptor: [Osteoarthritis] explode all trees 6007  #10 MeSH descriptor: [Arthritis] explode all trees 12880  #11 osteoarthrit* or osteoartrit* or osteo-arthrit* or osteo-artrit* or arthriti* or artriti* or arthros* or artros* or osteoarthros* or osteoartros* or OA 32754  #12 #9 or #10 or #11 33659  #13 MeSH descriptor: [Disease Progression] explode all trees 6632  #14 MeSH descriptor: [Prognosis] explode all trees 138404  #15 MeSH descriptor: [Incidence] explode all trees 9266  #16 MeSH descriptor: [Risk] explode all trees 35143  #17 increas* or worse* or deteriorat* or caus* or predict* or prognos* or precipitat* or inciden* or frequen* or rate* or occurr* 680198  #18 #13 or #14 or #15 or #16 or #17 722612  #19 #8 and #12 and #18 1660  #20 MeSH descriptor: [Pediatrics] explode all trees 607  #21 Infan* or newborn* or new-born* or perinat* or neonat* or baby* or babies or toddler* or minors* or boy or boys or boyhood or girl* or kid or kids or child* or adolescen* or juvenil* or youth* or teen* or "under*age*" or pubescen* or pediatric* or paediatric* or peadiatric* or school* or prematur* or preterm* 315893  #22 #20 or #21 315893  #23 #19 not #22 1137 |
| --- |
