# Supplementary material for: Risk factors for the progression of finger interphalangeal joint osteoarthritis: a systematic review
Source: Rheumatol Int. 2020 Aug 24;40(11):1781–92. doi: 10.1007/s00296-020-04687-1 (PMC7519919; doi:10.1007/s00296-020-04687-1)
Supplement: Supplementary file 2 — Supplementary file2 (DOCX 17 kb) [file 296_2020_4687_MOESM2_ESM.docx]

**Modified Quality in Prognostic Studies (QUIPS) tool**

| **Biases** | **Issues to consider for judging overall rating of "Risk of bias"** | **Study Methods & Comments** | **Rating of reporting** | **Rating of "Risk of bias"** |
| --- | --- | --- | --- | --- |
| Instructions to assess the risk of each potential bias: | These issues will guide your thinking and judgment about the overall risk of bias within each of the 6 domains. Some 'issues' may not be relevant to the specific study or the review research question. These issues are taken together to inform the overall judgment of potential bias for each of the 6 domains. | Provide comments or text excerpts in the white boxes below, as necessary, to facilitate the consensus process that will follow. | Rate the adequacy of reporting as yes, partial, no or unsure. | Rate potential risk of bias for each of the 6 domains as High, Moderate, or Low considering all relevant issues |
| **1. Study Participation** | **Goal: To judge the risk of selection bias (likelihood that relationship between *PF* and *outcome* is different for participants and eligible non-participants).** |  |  |  |
| *a) Source of target population* | The source population or population of interest is adequately described for at least 1 key characteristics |  |  |  |
| *b) Method used to identify population* | The sampling frame and recruitment are adequately described, including methods to identify the sample sufficient to limit potential bias (number and type used, e.g., referral patterns in health care) |  |  |  |
| *c) Recruitment period* | Period of recruitment is adequately described |  |  |  |
| *d) Place of recruitment* | Place of recruitment (setting and geographic location) are adequately described |  |  |  |
| *e) Inclusion and exclusion criteria* | Inclusion and exclusion criteria are adequately described (e.g., including explicit diagnostic criteria or  “zero time” description). |  |  |  |
| *f) Adequate study participation* | There is 80% participation in the study by eligible individuals |  |  |  |
| *g) Baseline characteristics* | The baseline study sample (i.e., individuals entering the study) is adequately described for at least 1 key characteristics |  |  |  |
| **High risk if: more than half are no or unsure, Low risk if: all are yes, Moderate risk if: any other combination** | |  |  |  |
| **2. Study Attrition** | **Goal: To judge the risk of attrition bias (likelihood that relationship between *PF* and *outcome* are different for completing and non-completing participants).** |  |  |  |
| *a) Proportion of baseline sample available for analysis* | Response rate (i.e., proportion of study sample completing the study and providing outcome data) is 80% |  |  |  |
| *b) Attempts to collect information on participants who dropped out* | Attempts to collect information on participants who dropped out of the study are described. |  |  |  |
| *c)Reasons and potential impact of subjects lost to follow-up* | Reasons for loss to follow-up are provided. |  |  |  |
| *d) Outcome and prognostic factor information on those lost to follow-up* | Participants lost to follow-up are adequately described for baseline characteristics |  |  |  |
|  | There are no important differences between baseline characteristics and outcomes in participants who completed the study and those who did not. |  |  |  |
| **High risk if: more than half are no or unsure, Low risk if: all are yes, Moderate risk if: any other combination** | |  |  |  |
| **3. Prognostic Factor Measurement** | **Goal: To judge the risk of measurement bias related to how PF was measured (differential measurement of PF related to the level of outcome).** |  |  |  |
| *a)Definition of the PF* | A clear definition or description of 'PF' is provided (e.g., including dose, level, duration of exposure, and clear specification of the method of measurement). |  |  |  |
| *b) Valid and Reliable Measurement of PF* | Method of PF measurement is adequately valid and reliable to limit misclassification bias (e.g., may include relevant outside sources of information on measurement properties, also characteristics, such as blind measurement and limited reliance on recall). |  |  |  |
|  | Continuous variables are reported or appropriate cut-points (i.e., not data-dependent) are used. |  |  |  |
| *b) Method and Setting of PF Measurement* | The method and setting of measurement of PF is the same for all study participants. |  |  |  |
| *d) Proportion of data on PF available for analysis* | All of the study sample has complete data for PF variable. |  |  |  |
| **High risk if: more than half are no or unsure, Low risk if: all are yes or no known prognostic factors, Moderate risk if: any other combination** | |  |  |  |
| **4. Outcome Measurement** | **Goal: To judge the risk of bias related to the measurement of outcome (differential measurement of outcome related to the baseline level of PF).** |  |  |  |
| *a) Definition of the Outcome* | A clear definition of how IPJ OA is measured is provided, including duration of follow-up and level and extent of the outcome construct. |  |  |  |
| *b) Valid and Reliable Measurement of Outcome* | The method of outcome measurement used is adequately valid and reliable to limit misclassification bias (Those assessing OA are blind to prognostic factor measurement |  |  |  |
| *c) Method and Setting of Outcome Measurement* | The method and setting of outcome measurement is the same for all study participants. |  |  |  |
| **High risk if: more than half are no or unsure, Low risk if: all are yes, Moderate risk if: any other combination** | |  |  |  |
| **5. Statistical Analysis and Reporting** | **Goal: To judge the risk of bias related to the statistical analysis and presentation of results.** |  |  |  |
| *a) Presentation of analytical strategy* | There is sufficient presentation of data to assess the adequacy of the analysis. |  |  |  |
| *b) Model development strategy* | The strategy for assessing the prognostic factor is appropriate and is based on a conceptual framework or model. |  |  |  |
|  | The selected statistical tool is adequate for the design of the study. |  |  |  |
| *c) Reporting of results* | There is no selective reporting of results. |  |  |  |
